# Optimization of Laponite Nanogel with Curcumin Incorporation: A Quality by Design Approach

**DOI:** 10.3390/gels11090677

**Published:** 2025-08-24

**Authors:** Jing Li, Xiangfeng Kong, Hongxia Chen, Mengqiu Lu, Xiaochang Liu, Lijie Wang

**Affiliations:** School of Pharmacy, Shenyang Medical College, No. 146 Huanghe North Street, Shenyang 110034, China; defghijklmn@163.com (J.L.);

**Keywords:** design of experiment, quality by design, laponite nanogel, curcumin, optimal formulation, stability

## Abstract

Nanogel is administered via various routes to overcome physiological barriers and achieve the desired therapeutic effect in vivo. However, developing a stable nanogel system is required to retain its therapeutic efficacy after storage. Therefore, a nanogel system composed of inorganic material (Laponite) was developed using Quality by Design (QbD) and Design of Experiments (DoE), using curcumin (CUR) as a model drug. Through a comprehensive literature review, single-factor experiments and Box–Behnken Design (BBD) experiments, we identified the CQAs and critical process parameters (CPPs), ultimately obtaining the optimal formulation. The DL, EE, Ps and PDI were determined as the CQAs and the optimal formulation was successfully prepared (LAP:CUR:TPGS = 6:2:36.6; mg;10 mL). FTIR, DSC and TEM analyses confirmed the successful loading of CUR, with a Ps in100nm, exhibiting biphasic drug release characteristics and maintaining stability for 28 days at 4 °C. QbD combined with DOE successfully facilitated stable CUR-TPGS-LAP nanogels. This study helps to better understand the critical factors in the development of nanogels and lays the foundation for the future integration of AI technology to promote a “first-time-right” drug formulation for future AI-promoted ‘one-stop’ drug formulation development model.

## 1. Introduction

Nanotechnology has the potential to enhance the in vivo distribution of drugs, promote transmembrane transport and achieve targeted delivery [1]. Among these, inorganic nanogels demonstrate remarkable stability, diverse physicochemical properties, and modifiable characteristics, making them highly valuable in bioimaging and drug delivery [2]. Laponite (LAP) is a unique layered nanogel characterized by a microstructure composed of disk-shaped particles with charged surfaces on both sides [3]. With its antibacterial properties, biocompatibility, safety and adsorption, as well as its thickening and thixotropic characteristics, LAP serves as an excellent pharmaceutical excipient [4].

LAP achieves universal loading of ionic, polar and non-polar drugs through multi-mechanism and multi-space loading modes [5,6]. It can assemble polymers without chemical modification, retaining natural biocompatibility, making it an ideal drug delivery platform [7,8,9]. This assembly of LAP’s functionality achieves programmability within its structure, enhancing drug loading capabilities while facilitating intelligent stimulus-responsive drug release [10,11,12]. The rapid research and development of stable LAP polymer nanogels not only avoids the high energy consumption and time-consuming nature of traditional methods but also accelerates the safe clinical translation of innovative therapies.

**Figure 1:**
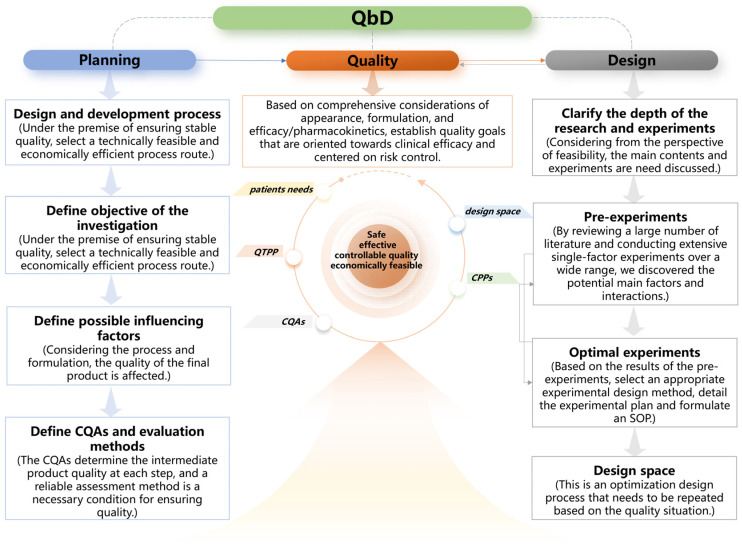
Summary of the general workflow used for the QbD of a DoE.

The selection of excipients, formulation optimization and preparation processes are crucial determinants in the development of LAP nanogels. These factors directly influence particle size (Ps), polydispersity index (PDI), drug release rate, physicochemical stability and bioavailability. The implementation of the Quality by Design (QbD) approach is critically important in the field of nanogel development. By employing systematic experimental design and risk management strategies, QbD enhances research and development efficiency while improving product quality and the feasibility of industrial-scale production [13]. QbD is an advanced pharmaceutical concept recognized by the FDA [14], with its core principle being the integration of quality into the entire process of product and process design, rather than relying solely on final testing. Its goal-oriented process encompasses determining critical quality attributes (CQAs) based on predefined quality target product profiles (QTPPs); identifying critical material attributes (CMAs) and critical process parameters (CPPs) through risk assessment and experimentation; establishing a design space and formulating a control strategy to ensure process robustness; and ultimately implementing continuous improvement to manage the lifecycle. QbD integrates tools such as risk assessment and design of experiments (DoE) to proactively manage variability rather than eliminate it, ensuring that products consistently meet quality requirements. The general workflow for the combined use of the QbD and DOE is illustrated in Figure 1. This approach significantly enhances R&D efficiency, ensures quality, reduces risks and accelerates translation, making it a core strategy in modern formulation development [15,16,17,18].

DoE serves as a fundamental tool for executing the QbD concept [19,20,21]. In contrast to traditional experiments, DoE enables a comprehensive evaluation of the effects of excipients and process parameters with significantly fewer experimental runs. Within the QbD framework, DoE is essential for scientifically determining CMAs and CPPs, establishing a robust design space and ultimately facilitating a reliable quality control strategy [22].

This study aims to prepare stable curcumin LAP nanogel with an average diameter of 100–200 nm by employing the principles of QbD in our experimental framework. This article applies QbD principles and utilizes Minitab software (version 19, Minitab, Inc.) to illustrate the optimization process of polymer nanoparticle formulation. Furthermore, we implement the Box–Behnken design for optimization to achieve a more stable formulation. These findings are intended to provide valuable insights into the optimization of inorganic nanoparticle formulations for poorly soluble drugs.

## 2. Results and Discussion

### 2.1. Initial Identification of QTPP, CQA and CPP Elements

**Figure 2:**
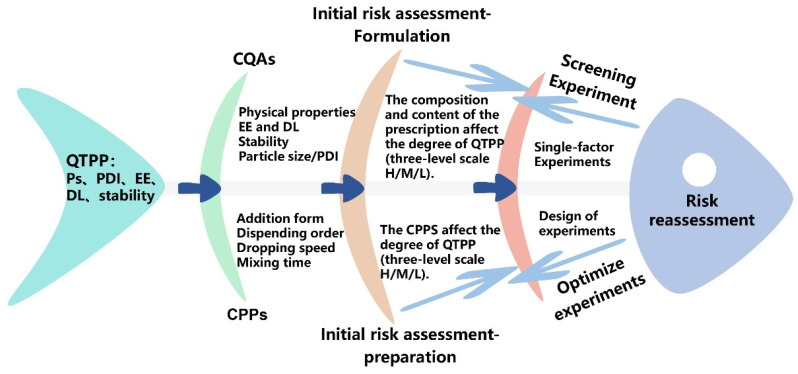
Ishikawa diagram of the QTPP and the related factors.

In this study, we identified the Ps, PDI, DL, EE and stability as the QTPP of CUR-LAP-TPGS. Table 1 presents the basis for determining the CQAs in this study. In addition to evaluating the quality of the formulation itself, such as Ps, PDI, DL and EE, the stability was also investigated as a CQA. This investigation is crucial to ensure that the nanogel exerts its therapeutic effects while preventing drug precipitation and sedimentation. Furthermore, considering the influence of the preparation process on the properties of excipients and the overall quality of the product, we developed a relationship diagram that connects the QTPP, CQAs and CPPs, as illustrated in Figure 2.

### 2.2. Pre-Experiment Results and Risk Re-Assessment

**Figure 3:**
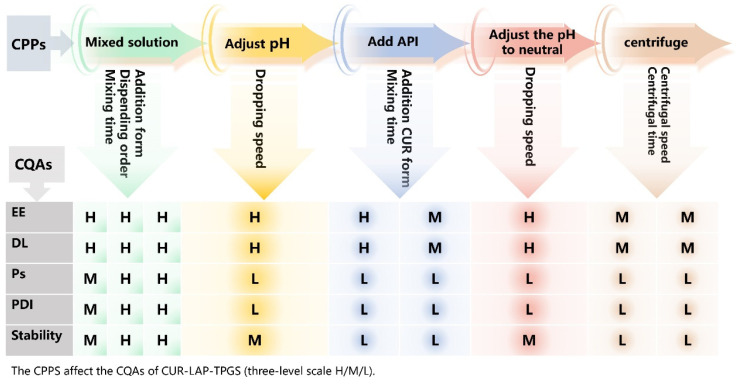
Initial risk assessment of CPPs.

**Figure 4:**
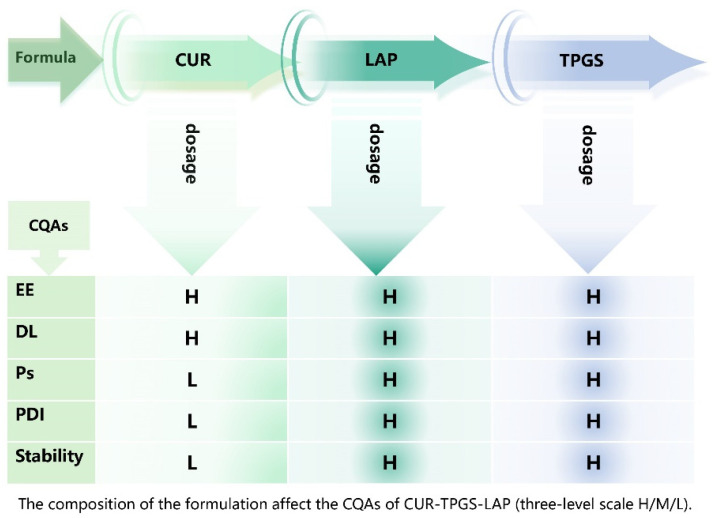
Initial risk assessment of prescription.

The initial risk assessment of the process and formulation composition is illustrated in Figure 3 and Figure 4. Based on this assessment, the effects of adding versus not adding components, as well as different addition forms on Ps, PDI, DL and EE were compared. The results are presented in Table 2. When the stirring time, the dripping sequence of LAP and TPGS and the pH value were kept constant, pre-mixing TPGS with LAP prior to the addition of CUR powder significantly reduced the size of the nanoparticles. This reduction is primarily attributed to the increased density of TPGS due to pre-mixing, which enhanced the dissolution of CUR and facilitated the formation of smaller particles [9].

The addition of TPGS significantly affected Ps, PDI, DL and EE. This effect is primarily due to TPGS, as a common non-ionic surfactant, enhancing the solubility of CUR, increasing CUR concentration and promoting its encapsulation in the LAP [4,23]. The addition of LAP in powder form also significantly impacts DL, EE, PS and PDI. When LAP is introduced into the system in powder form, it gradually swells and dissolves, subsequently assembling with TPGS into layered composite nanogels. The drug-loading capacity of LAP is influenced by factors such as the charge of the drug and Laponite, as well as the concentrations of the drug and Laponite [4]. During this slow assembly process, CUR gradually combines with TPGS, increasing its solubility, and ultimately forms nanogels with a uniform size and high encapsulation.

The final determined process involves dissolving the specified amount of TPGS in 9 mL of distilled water, stirring until fully dissolved. Subsequently, LAP powder is added and the mixture is stirred for 1 h. Following this, 1 mL of NaOH solution (pH = 13) is incorporated and the mixture is stirred thoroughly. CUR powder is then added, and stirring continues for an additional hour. The pH of the solution is adjusted to neutral using HCl (0.4 M) solution, followed by stirring for 15 min. The mixture is then centrifuged at 4000 rpm for 10 min, and the supernatant is collected to obtain the final product.

The results of single-factor experiment regarding the effects of CUR, TPGS and LAP on Ps, PDI, DL and EE are illustrated in the Supplementary Materials. As illustrated in Figure S1, increasing the CUR dosage resulted in an initial decrease in DL followed by an increase, and the same trend was observed for EE. And the PDI first increases and then decreases, however, these overall changes were not statistically significant (*p* > 0.05). In contrast, the Ps exhibited a gradual increase. LAP facilitates drug loading through mechanisms such as ion exchange or surface adsorption. As the quantity of the drug increases, additional drug molecules are either inserted into the interlayers or adsorbed onto the surface, resulting in an expansion of the interlayer spacing or an increase in the hydrated volume of the particles [24,25].The surface dynamic adsorption equilibrium characteristics of Laponite, along with the steric hindrance effect of TPGS, contribute to the stability of DL, EE and PDI by inhibiting particle aggregation [24,26].

The impact of LAP on Ps, PDI, DL and EE is similar to that of CUR. This stability can be attributed to the dynamic adsorption equilibrium of LAP under a constant CUR amount, along with the colloidal protection provided by TPGS, which results in minimal changes in DL, EE and PDI [25]. However, excessive LAP may cause reassembly through electrostatic interactions, leading to an increase in Ps and the formation of larger nanogels [27].

The EE, Ps and PDI of this formulation exhibited no significant changes with the increasing concentration of TPGS (*p* > 0.05) (Figure S3). This stability can be attributed to the spatial hindrance created by TPGS, which plays a stabilizing role [28]. However, when TPGS is combined with LAP for drug loading, its molecular chains obscure certain active sites of Laponite, directly reducing the effective area available for drug adsorption and consequently decreasing the DL. Nevertheless, the maximum drug adsorption capacity of LAP can still maintain a high adsorption efficiency even under saturation conditions constant CUR, with the EE remaining unchanged [25].

Based on the influences of CUR, LAP and TPGS in the formulation, we established the ranges for the three factors in the DOE experiment according to the pre-experimental results: CUR: 2–10 mg; LAP: 2.5–10 mg; TPGS: 15–45 mg; 10 mL system.

### 2.3. Results of DoE

Based on the results of a single-factor experiment, a significance analysis was performed on the four response values: DL, EE, Ps and PDI. The findings are detailed in the Supplementary Material. The amount of CUR significantly influenced DL and EE, exhibiting a relatively large range and a nonlinear effect (Figures S4 and S5). However, for Ps and PDI, which were maintained at constant values, the effect of CUR was not significant. Notably, when another influencing factor was adjusted, Ps and PDI remained unchanged (Figures S4 and S5). Similarly, when evaluating the effects of LAP and TPGS on DL, EE, Ps and PDI, we found that TPGS had a relatively significant impact on Ps compared to CUR and LAP, as referenced in Figures S4–S7. Although LAP influenced PDI, the values of PDI exhibited satisfactory dispersity (PDI < 0.3) [29,30] within the selected ranges of CUR, LAP and TPGS, thereby fulfilling the uniformity requirements for nano-formulations. Within these specified ranges, the effect of LAP on the four response values was found to be insignificant. Consequently, to meet the comprehensive requirements for DL, EE, Ps and PDI, CUR, LAP and TPGS were identified as the critical factors for optimization.

**Figure 5:**
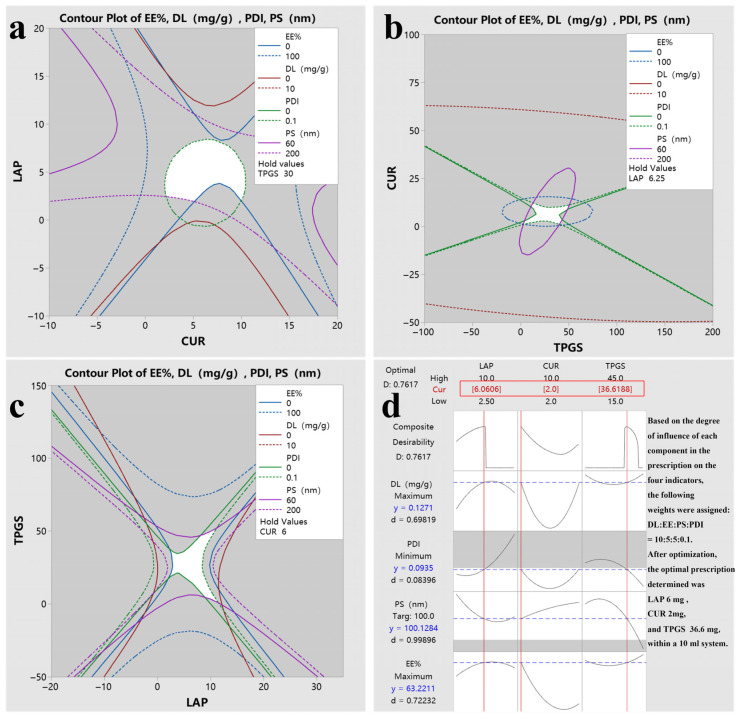
The overlapping contour plot of each response value and optimal prescription. (**a**) Contour plot of LAP and CUR. (**b**) Contour plot of CUR and TPGS. (**c**) Contour plot of TPGS and LAP. (**d**) Optimal prescription.

Figure 5a–c illustrate the overlapping contour plots that depict the effects of DL, EE, Ps and PDI across the following three factors: CUR, TPGS and LAP. The white regions in these plots signify the optimal factor areas identified after selecting the desired effect values. Consequently, response optimization was conducted based on these findings.

According to the optimization objectives and weights of the response values, the optimization results of the response values are shown in Figure 5d. The results indicate that the Composite Desirability exceeds 0.7, which confirms the reliability of the proposed plan [31]. The desirability values for DL, EE and Ps, which possess higher weight ratios, are also close to or exceed 0.7, further demonstrating the robustness of the optimized plan. Although the desirability of PDI is relatively low, with values consistently at 0.3, this suggests a good, uniform dispersion of the nanogels. Moreover, due to the minor weight of PDI, its desirability value can be considered negligible. In Figure 5d, ‘y’ represents the predicted value, which closely approximates the target set. The desirability values for DL, EE and Ps are all approximately 0.7, indicating that the predictive value is relatively reliable. Therefore, the optimal formulation is identified as LAP:CUR:TPGS = 6:2:36.6 (mg; in a 10 mL system).

### 2.4. The Characterization Results of the Optimal Prescription

The successful incorporation of CUR into CUR-TPGS-LAP was confirmed through the structural characteristics observed in TEM images, the shifts and intensity variations in relevant peaks in FTIR spectra and the elimination of the CUR melting peak in DSC analysis. Furthermore, the effective encapsulation of CUR within CUR-TPGS-LAP was substantiated by the absence of burst release in vitro and the consistent stability of Ps and PDI over a 28-day period.

#### 2.4.1. TEM

**Figure 6:**
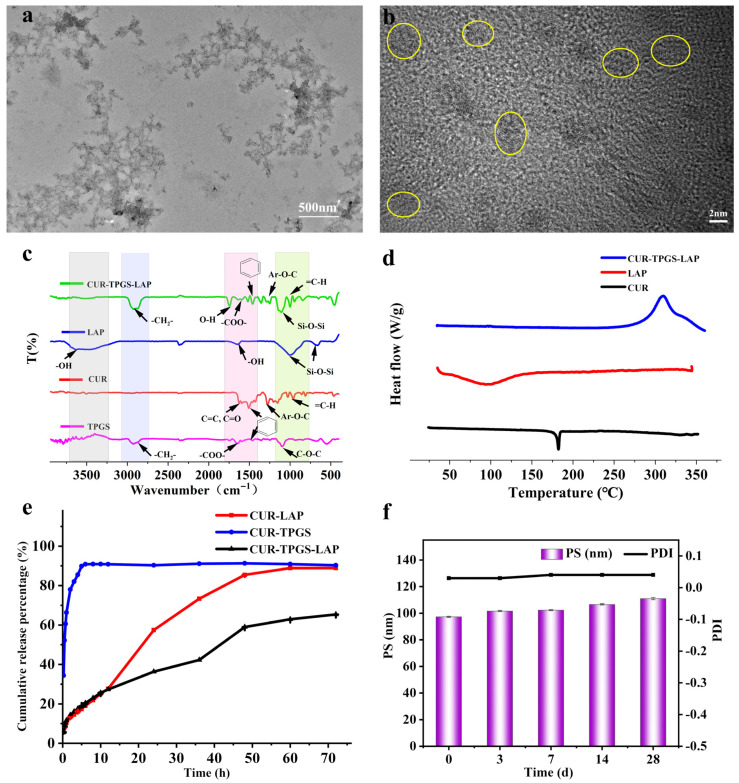
The characterization of the optimal CUR-TPGS-LAP. (**a**) TEM image and (**b**) high-resolution TEM images. (**c**) FTIR spectrum. (**d**) DSC curve. (**e**) In vitro drug release. (**f**) The changes in Ps and PDI after storage at 4 °C for 3 d, 7 d, 14 d and 28 d (mean ± S.D., n = 3, *p* > 0.05).

The optimal formulation of CUR-LAP-TPGS is clearly illustrated in the TEM images (Figure 6a,b). The result indicates that the overall morphology of the nanoparticles is uniform, with no visible crystalline particles or sharp angled structures. This figure presents a typical morphology of an interwoven nano-disk network, which primarily arises from the octahedron-like configuration formed by the interaction between the positively charged edges and the negatively charged interlayer regions of LAP [4]. The unit size of this network structure is approximately 100 nm, which is smaller than the particle size measured by the Malvern particle size analyzer. This discrepancy is mainly attributed to the presence of nanoscale particles and hydrophilic groups on the surface, resulting in an increase in the hydrated particle size [32]. As shown in Figure 6b, distinct lattice fringes are observable in the monolayer structure of LAP; although the lattice resolution is limited, it is sufficient to demonstrate that the structural integrity of LAP is preserved.

#### 2.4.2. FTIR

The functional groups and structural changes in CUR-TPGS-LAP were detected at the molecular level by FTIR. The FTIR spectra of CUR, LAP and CUR-TPGS-LAP nanogels are shown in Figure 6c. The FTIR spectrum of LAP shows that its characteristic vibration bands have a broad and strong absorption peak at about 3000–4000 cm^−1^, mainly attributed to the -OH stretching vibration of interlayer adsorbed water molecules, and a peak at 1623 cm^−1^ corresponding to the bending vibration of interlayer -OH. The strongest absorption peak at about 1010–1040 cm^−1^ is attributed to the antisymmetric stretching vibration of Si-O-Si, and the position and shape of this peak are very sensitive to the structure of the silicate framework (such as the order of tetrahedral sheets and interlayer interactions) in LAP [33,34]. The peak at 670–690 cm^−1^ is attributed to the symmetric bending vibration of Si-O-Si.

The most characteristic peaks of CUR’s structure is a strong peak at 1650–1620 cm^−1^, attributed to the vibration of conjugated enone systems (coupled with C = C and C = O), and a peak at 960 cm^−1^ attributed to the out-of-plane bending vibration of the trans alkene = C-H. The peaks at 1500–1600 in the spectrum indicate the presence of an aromatic ring in CUR. The peaks at 2950–2850 cm^−1^, 1430–1400 cm^−1^ and 1280–1260 cm^−1^ in the spectrum correspond to the vibration peaks of C-H, CH_3_ and Ar-O-C, jointly indicating the presence of a methoxy group in CUR [35].

TPGS shows a strong vibration during hydrophobic -CH_2_- stretching at 2926 cm^−1^, a stretching vibration of COO^−^ at 1665 cm^−1^ and a stretching vibration of C-O-C at 1100 cm^−1^ in the infrared spectrum [36,37]. In the FTIR spectrum of CUR-TPGS-LAP, the -CH_2_- stretching vibration peak of TPGS at 2926 cm^−1^ remains evident. Following CUR loading, the -OH of LAP and the COO^−^ of TPGS exhibit shifts, indicating the potential formation of hydrogen bonds between them. Upon curcumin loading, the -OH of LAP and the COO^−^ of TPGS undergo shifts, further suggesting the possible formation of hydrogen bonds between these components. The shift in the C = O bond in CUR to a lower wavenumber, accompanied by the weakening of the aromatic ring peaks, suggests that CUR is encapsulated. Additionally, the change in the Si-O-Si absorption peak of LAP, observed at 1010–1040 cm^−1^, indicates alterations in the order of tetrahedral sheets and interlayer interactions in the silicate framework, thereby indicating the successful loading of CUR-TPGS in LAP [9].

#### 2.4.3. DSC

As illustrated in Figure 6d, the most prominent characteristic of the DSC curve for CUR is a sharp endothermic peak, which corresponds to its melting point within the range of 175 °C to 185 °C [38]. LAP contains both structural water and interlayer water and exhibits a broad endothermic peak, primarily occurring around 100 °C. This peak corresponds to the gradual removal of adsorbed water (interlayer water) and some structural water [39]. Notably, the CUR-TPGS-LAP shows the complete disappearance of CUR’s characteristic melting peak when compared to the raw drug, exhibiting no melting behavior and suggesting that CUR exists in an amorphous or molecularly dispersed state. Furthermore, the CUR-TPGS-LAP reveals an exothermic decomposition peak of TPGS within the range of 300° C to 350° C, which may suggest the thermal decomposition/oxidation of TPGS.

#### 2.4.4. In Vitro Drug Release

In Figure 6e, the CUR-TPGS exhibits rapid release within one hour; however, sustained-release behavior is not observed. Conversely, the CUR-LAP demonstrates a slow release over a period of 12 h. The CUR-TPGS-LAP, on the other hand, shows a relatively rapid release within the first four hours, followed by a slower release between four and forty-eight hours, indicating a biphasic drug-release characteristic. Initially, the free drugs adsorbed on the surface and interlayers of the LAP plate are preferentially released, followed by the CUR encapsulated within the micelles, which releases CUR at a relatively slower rate. By integrating two distinct drug release modes—rapid release and sustained release—this approach transcends the limitations of traditional formulations, facilitating both rapid onset and prolonged maintenance. This strategy effectively addresses the time lag effect, aligns with physiological rhythms and significantly enhances both efficacy and safety.

#### 2.4.5. Stability

In Figure 6f, the Ps and PDI of CUR-TPGS-LAP did not exhibit significant changes over a 28-day period (*p* > 0.05). In CUR-TPGS-LAP, LAP effectively prevents the dissociation of CUR-TPGS through interlayer confinement, maintaining micelle stability via van der Waals forces. Additionally, TPGS contributes to steric hindrance, exerting a stabilizing effect that enhances the anti-aggregation capability of CUR-TPGS-LAP [28].The Mg-OH and Si-OH groups at the edges of the LAP layer can form hydrogen bonds and ionic bonds (Mg^2+^ and -COO^−^) with the -COO^−^ groups of TPGS, effectively covering and passivating the high-energy active sites [25]. This reduces non-specific adsorption of micelles at the interface. LAP’s ability to stabilize micelles offers significant potential for biphasic release, which is closely related to the biphasic release behavior exhibited by CUR-TPGS-LAP in vitro, as discussed in Section 2.4.4. The current stability data represent preliminary observations conducted at 4 °C for a duration of 28 days. Future research should aim to validate these findings across extended durations and in relevant clinical storage environments.

## 3. Conclusions

In this study, we developed a nanogel system composed of LAP-TPGS using a combined approach of QbD and DoE, with curcumin (CUR) as the model drug. This research investigates the construction steps of LAP nanogel and examines the impact of the system’s CQAs on formulation stability. The successful loading of CUR was verified through FTIR, DSC and TEM. Additionally, the stability of the nanogel was assessed using in vitro release and stability studies. Overall, our study employs a combined strategy of QbD and DoE to provide a novel framework for the development of nanogel systems, and consolidates LAP as a viable drug delivery system. Future in-depth research on the drug loading and release mechanisms of LAP nanogels will demonstrate significant therapeutic potential at various administration sites.

Although published work can support the optimization of poorly soluble drug nanogels, further research is necessary for certain new materials or newly combined materials. A different DoE should be employed to obtain more representative data for the development of nanogel formulations. Starting from a design perspective, it is crucial to select the variables that have the greatest impact on CQAs’ target quality and to understand how they influence these CQAs. This understanding is vital for ensuring the safety and quality of drug development.

The combination of DoE and QbD is significantly importance in the development of drug delivery. Firstly, it reduces trial and error through DOE, significantly enhancing the efficiency of the development of new formulations. Secondly, based on mathematical models and statistical calculations, the design space provides a scientifically flexible control range for production processes, thereby providing reliable assurances for large-scale production. Finally, this development model ensures the precise attainment of product quality attributes, thereby accelerating regulatory review and market entry. DoE combined with QbD will deeply integrate AI technology into the development of new formulations, creating a closed loop of “digital design–intelligent optimization–experimental verification”. Furthermore, when combined with advanced-process analytical technologies, this approach will further promote the achievement of a “one-time success” development model. The potential prospects of a combined DoE and QbD research and development model in formulation development are promising.

In prescription development research, the integration of a physical property database of raw materials with molecular simulation experiments facilitates the intelligent matching of excipients, reducing the screening period. In process and prescription optimization, the use of reasonable experimental designs can reduce the number of experiments, construct a suitable design space and integrate mathematical models to ensure the construction of complex formulations. In quality control, during process scaling-up, advanced process analytical technologies can be combined for the dynamic control of CPPs. In drug regulation and market entry, the optimized models, design space and risk control data from QbD can be combined to ensure drug quality, accelerate the review process and reduce the risk of clinical failure.

## 4. Materials and Methods

### 4.1. Materials

Curcumin (CUR) was acquired from Dalian Meilun Biotechnology Co., Ltd. (Dalian, China). LAP was sourced from Nanjing Baiyike New Material Technology Co., Ltd. (Nanjing, China), while vitamin E polyethylene glycol succinate (TPGS) was obtained from McLean Biochemical Technology Co., Ltd. (Shanghai, China). Tween 80 was procured from Damao Chemical Reagent Factory Co., Ltd. (Tianjin, China). Additionally, sodium hydroxide, ethanol, potassium dihydrogen phosphate and hydrochloric acid were also purchased from Damao Chemical Reagent Factory Co., Ltd. (Tianjin, China).BT-Zeta100 analyzer (Dandong Best Technology Co., Ltd., Dandong, China), Transmission electron microscopy JEM-2100F TEM measurements (JEOL Co., Ltd., Tokyo, Japan), Fourier transform infrared (FT-IR) spectrometer (Beijing Beifen Ruili Analytical Instru-ment Co., Ltd., Beijing, China), Differential Scanning Calorimetry HSC-4 (Beijing Hengjiu Experimental Equipment Co., Ltd., Beijing, China), Constant Temperature Shaker Guohua Enterprise SHA-C (Guohua Enterprise Management Group Co., Ltd., Beijing, China), and data analysis was performed using SPSS version 26.0 (IBM, Armonk, New York, NY, USA). 

### 4.2. Methods

#### 4.2.1. Process of the QbD Design

The QbD approach to formulation closely mirrors the product development process involved in creating pharmaceutical preparations, emphasizing formulation-related factors and quality [40,41,42]. This process generally encompasses the determination of the product’s quality profile, identification of critical quality attributes, establishment of critical process parameters, preliminary risk assessment, pre-experimentation activities, experimental design and risk re-evaluation. Figure 1 illustrates the specific process.

#### 4.2.2. Determine Elements of the QTPP

QTPP encompasses essential elements such as dosage form, mechanism of action, route of administration, specifications, stability, content, hazardous substances, dissolution, residual solvents, microorganisms and packaging systems [43,44,45]. These components constitute the foundational aspects of the QbD methodology. The QTPP serves as a forward-looking summary of the ideal quality characteristics of a product, guiding the control of the final product’s quality. We searched the PubMed database and identified the QTPP for this study using the following keywords: laponite, nanogel, QbD and DOE.

The QTPP serves as a forward-looking summary of the ideal quality characteristics of a pharmaceutical product, guiding the control of the final product’s quality. In our study, we conducted a search in the PubMed database to identify the QTPP using the following keywords: laponite, nanogel, QbD and Design of Experiments (DOE).

#### 4.2.3. Determine Elements of the CQAs

CQAs refer to the specific physical, chemical, biological or pharmaceutical properties of finished materials. These attributes are typically derived from the QTPP and supplemented with prior knowledge [46,47,48,49]. CQAs are identified through risk assessment and are used to guide product and process development. During the initial stages of development, CQAs and their target ranges should be established through a comprehensive risk assessment. This process should utilize prior knowledge, the relevant literature and carefully designed experiments to ensure robustness and reliability.

#### 4.2.4. Risk Assessment of CPPs

In the development of nanogels, various process factors significantly impact the quality of the final product. These process parameters, including stirring speed, stirring time and solvent addition rate, also play important roles in determining these CQAs [50,51]. In this experiment, we evaluate the risks associated with CQAs and their CPPs. This section employs a three-level scale, categorized as ‘High’ (H), ‘Medium’ (M) or ‘Low’ (L), to characterize the risk relationships between these parameters.

#### 4.2.5. Risk Assessment of Formulation

The composition and ratio of the formulation can significantly influence the product. During the risk assessment process, it is essential to evaluate the interdependencies and impacts of the quality attributes, CQAs and CPPs. In this study, we summarized the influence of formulation composition and CQAs based on the results of preliminary experiments and employed a three-level rating system (High (H)/Medium (M)/Low (L)) for the continuous assessment of risk factors.

##### Design of Experiments (DoE)

Based on the results of the single-factor investigation (Supplementary Materials), this study employed Box–Behnken Design (BBD) in conjunction with Response Surface Methodology (RSM) to optimize the formulation. Through the single-factor experiments, three critical variables were identified: the amounts of LAP, CUR and TPGS (Table 3). The indicators, including DL (Y1, mg/g), EE (Y2, %), Ps (Y3, nm) and PDI (Y4), were established.

The BBD examined the effects of the three critical factors on the four indicators. The experimental design comprised 15 groups (12 factorial combination points and 3 center points). The factor levels are detailed in Table 3: LAP (X1): 2.5 mg, 6.25 mg and 10 mg, designated as levels −1, 0 and 1, respectively; CUR (X2): 2 mg, 6 mg and 10 mg, also designated as levels −1, 0 and 1; TPGS (X3): 15 mg, 30 mg and 45 mg, similarly designated as levels −1, 0 and 1 (Table 3). The critical factor combinations for the 15 designed prescription optimizations are specified in columns X1, X2 and X3 of Table 4. Subsequently, random experiments were conducted following the preparation method for CUR-TPGS-LAP, corresponding to the 15 experimental groups. Four indicators were evaluated, which were measured sequentially after the completion of the prescription preparation. The measured results are recorded in columns Y1, Y2, Y3 and Y4 of Table 4.

For the four indicators across the 15 experimental groups, optimization criteria were established based on practical requirements. DL and DE were set as maximization criteria, Ps was set at 100 nm and PDI was designated as the minimization criterion. In this study, due to the four indicators optimization, weights were utilized to achieve a global satisfaction of multiple objectives, with the weights specified as DL:EE:Ps:PDI = 10:5:5:0.1. Finally, based on the established criterions, Minitab software was employed to fit each factor and indicator, generate the factor effect plot (Supplementary Materials) and produce the response surface, contour plot and optimization results to determine the optimal formulation.

##### Characterization of the CUR -TPGS-LAP

Particle Size and Zeta Potential

The Ps and PDI of the samples were measured using a BT-Zeta100 analyzer (Dandong Best Technology Co., Ltd., Dandong, China), with each sample analyzed in triplicate. The EE of curcumin was determined using a modified method [9]. Specifically, 0.5 mL of the supernatant was collected from the centrifuged sample and mixed with 9.5 mL of chloroform. The mixture was stirred overnight, after which the lower phase was collected, and the absorbance was measured at 427 nm. The entrapment efficiency (EE) was established based on the following equation (Equation (1)):

EE (%) = Curcumin in supernatant/Total added curcumin × 100(1)

TEM Measurements

The size, structure and morphology of the LAP nanogel were characterized using transmission electron microscopy (TEM). In this study, JEM-2100F TEM measurements (JEOL Co., Ltd., Tokyo, Japan) were utilized to prepare samples of CUR-TPGS-LAP. The samples were carefully deposited onto a copper mesh with an ultrathin carbon support film, allowed to dry naturally and subsequently examined for their morphology under an accelerated voltage of 200 kV.

FT-IR Spectroscopy Measurements

The infrared spectra of CUR, LAP and CUR-TPGS-LAP powders were obtained using the potassium bromide tableting method with a Fourier transform infrared (FT-IR) spectrometer (Beijing Beifen Ruili Analytical Instrument Co., Ltd., Beijing, China). For sample preparation, 1 to 2 mg of the solid sample was ground in a mortar and combined with 100 to 200 mg of dry KBr, which was further ground until a homogeneous mixture was obtained. A suitable amount of this mixture was placed into a mold and pressed into thin slices. The infrared spectrum was scanned over a wavelength range of 500 to 4000 cm^−1^.

DSC Measurements

In the Differential Scanning Calorimetry (DSC) (Beijing Hengjiu Experimental Equipment Co., Ltd. HSC-4, Beijing, China) test, appropriate quantities of CUR, LAP and CUR-TPGS-LAP powders were placed in an alumina crucible, while a blank alumina crucible was used as the reference. The scanning was conducted over a temperature range of 50 °C to 250 °C at a heating rate of 5 °C/min.

In vitro drug release studies

A 1.25% (W/V) Tween 80 solution in PBS (pH 7.4) was utilized as the dissolution medium, maintained at a temperature of 34 ± 0.5 °C with a rotation speed of 150 r/min inConstant Temperature Shaker Guohua Enterprise SHA-C (Guohua Enterprise Management Group Co., Ltd., Beijing, China) for the investigation of the release curves. A total of 2 mL of CUR-TPGS-LAP solution, prepared according to the optimal formulation, along with CUR-LAP and CUR-TPGS solutions of equivalent concentration, were pipetted into a pretreated dialysis bag (MW 20,000). The bag was securely tied with string and submerged in an Erlenmeyer flask containing 100 mL of the 1.25% (W/V) Tween 80 in PBS (pH 7.4) solution. The flask was agitated on a shaker, and samples were collected at 0.25, 0.5, 0.75, 1, 2, 3, 4, 5, 6, 8, 10, 12, 24, 36, 48, 60 and 72 h, with the dissolution medium replenished to maintain consistent temperature and volume. The absorbance of each sample solution was measured to calculate the in vitro release of CUR-LAP, CUR-TPGS and CUR-TPGS-LAP. The release curves for the three formulations were generated by plotting time (h) on the x-axis against the cumulative release percentage on the y-axis.

The stability of CUR-TPGS-LAP formulation

This study conducts an investigation into the stability of the optimally formulated CUR-TPGS-LAP, focusing on changes in Ps and PDI during storage at refrigeration temperature (4 °C). Samples were collected at 3, 7, 14 and 28 days and compared to freshly prepared formulations to evaluate their stability.

#### 4.2.6. Statistical Analysis

The data are presented as means ± standard deviation (SD) with a sample size of n = 3. Analyses were conducted using SPSS version 26.0 (IBM, Armonk, New York, NY, USA), and a One-way Analysis of Variance (ANOVA) was employed to determine the *p*-values. Significance levels were indicated as follows: *** *p* < 0.001, ** *p* < 0.01 and * *p* < 0.05.

## Figures and Tables

**Table 1:** Confirm the CQA_S_.

Quality Attributes	Is It a Critical Quality Attribute?	Justification
Physical properties	No	Physical properties such as color, odor and appearance are not CQA, as these factors have no direct relationship with the efficacy of the formulation.
Encapsulation efficiency (EE)/Drug Loading (DL)	Yes	A high encapsulation and drug loading are crucial for improving drug utilization, reducing waste, enhancing therapeutic efficacy, minimizing toxic side effects and lowering production costs. Therefore, they are regarded as CQA.
Particle size (Ps/Particle size distribution index (PDI)	Yes	Nanoparticles can more effectively pass through biological membranes within the appropriate nanoscale range, thereby improving solubility and absorption properties and enhancing the bioavailability of drugs. Therefore, they are regarded as CQA.
Stability	Yes	The stability of nanoparticles has a significant impact on the release of drugs and their efficacy. Therefore, it is regarded as a CQA

**Table 2:** The addition of excipients and API to the process.

Ingredient	State	DL (mg/g)	EE (%)	Ps (nm)	PDI
CUR	solution	0.01 ± 0.00	12.87 ± 0.04	2670.00 ± 1565.22	0.45 ± 0.06
	powder	0.01 ± 0.00	14.99 ± 0.11	1220.33 ± 319.28	0.40 ± 0.05
TPGS	−	0.004 ± 0.00	15.03 ± 0.04	339.67 ± 11.73	0.35 ± 0.10
	+	0.01 ± 0.00	22.83 ± 0.04	204.00 ± 6.16	0.26 ± 0.02
LAP	solution	0.01 ± 0.00	16.96 ± 0.07	138.33 ± 1.25	0.21 ± 0.01
	powder	0.02 ± 0.00	29.85 ± 0.14	118.67 ± 0.94	0.12 ± 0.01

**Table 3:** The level of factors in the prescription.

Factors (mg)	Levels		
	−1	0	1
X1	2.5	6.25	10
X2	2	6	10
X3	15	30	45

X1 is the mass of LAP in the prescription, X2 is the mass of curcumin and X3 is the mass of TPGS.

**Table 4:** Box–Behnken Design experiment.

Run Order	Factors						
	X1	X2	X3	Y1	Y2	Y3	Y4
1	6.25	10	45	0.14	13.57	128.0	0.041
2	2.50	2	30	0.06	30.75	223.0	0.023
3	10.00	6	15	0.03	4.23	206.0	0.059
4	2.50	6	45	0.06	10.58	48.4	0.032
5	6.25	2	45	0.17	85.90	46.6	0.028
6	10.00	2	30	0.09	44.37	121.0	0.336
7	6.25	6	30	0.06	9.97	169.0	0.035
8	6.25	6	30	0.06	9.92	141.0	0.043
9	6.25	2	15	0.15	75.56	107.0	0.151
10	10.00	6	45	0.05	8.88	103.0	0.045
11	6.25	6	30	0.06	9.92	143.0	0.025
12	2.50	6	15	0.05	8.94	167.0	0.037
13	6.25	10	15	0.05	5.16	76.5	0.03
14	10.00	10	30	0.10	9.83	202.0	0.318
15	2.50	10	30	0.12	11.47	172.0	0.034

X1 is the mass of LAP in the prescription, X2 is the mass of curcumin and X3 is the mass of TPGS. Y1 represents DL, Y2 represents EE, Y3 represents Ps and Y4 represents PDI.

## Data Availability

The original contributions presented in this study are included in the article/Supplementary Materials. Further inquiries can be directed to the corresponding author.

## Supplementary Materials

The following supporting information can be downloaded at: https://www.mdpi.com/article/10.3390/gels11090677/s1, The following supporting information explores the impact of each component in the prescription on Ps, DL, EEr and PDI., Figure S1: The influence of the quantities of CUR on the DL, Ps, EE and PDI, (mean ± S.D., n = 3); Figure S2: The influence of the quantities of LAP on the DL, Ps, EE and PDI, (mean ± S.D., n = 3); Figure S3: The influence of the quantities of TPGS on the DL, Ps, EE and PDI, (mean ± S.D., n = 3); Figure S4. The effects of CUR, LAP and TPGS on the DL in contour and surface plots; Figure S5. The effects of CUR, LAP and TPGS on the EE in contour and surface plots; Figure S6. The effects of CUR, LAP and TPGS on the Ps in contour and surface plots; Figure S7. The effects of CUR, LAP and TPGS on the PDI in contour and surface plots.

## Abbreviations

The following abbreviations are used in this manuscript:

QbD	Quality by Design
QTPP	Quality Target Product Profile
CQAs	Critical Quality Attributes
CPPs	Critical Process Parameters
DOE	Design of Experiments
CUR	Curcumin
TPGS	Vitamin E Polyethylene Glycol Succinate
LAP	Laponite
DL	Drug Loading
Ps	Particle Size
EE	Encapsulation Efficiency
PDI	Polydispersity Index
TEM	Transmission Electron Microscopy
FTIR	Fourier Transform Infrared Spectroscopy
DSC	Differential Scanning Calorimetry
