# Supplementary material for: Optimization of Laponite Nanogel with Curcumin Incorporation: A Quality by Design Approach
Source: Gels. 2025 Aug 24;11(9):677. doi: 10.3390/gels11090677 (PMC12469730; doi:10.3390/gels11090677)
Supplement: Supplementary file 1 [file gels-11-00677-s001.zip › gels-3792597-supplementary.pdf]

Supplementary Materials

Optimization of Laponite Nanogel with Curcumin Incorporation: A Quality by Design Approach

Jing Li, Xiangfeng Kong, Hongxia Chen, Mengqiu Lu, Xiaochang Liu and Lijie Wang *

School of Pharmacy, Shenyang Medical College, No. 146 Huanghe North Street, Shenyang 110034, China; defghijklmn@163.com (J.L.); k18242406211@163.com (X.K.); 17789677613@163.com (H.C.);
lumengqiu2589@163.com (M.L.); liuxiaochang1991@163.com (X.L.)
* Correspondence: wanglijie_a318@163.com or wanglijie@symc.edu.cn

Table of contents:

1. The Single factor Experiments S1
2. Effect of Control Factors on the Response S1
Figure S1. The influence of the quantities of CUR on the DL, Ps, EE and PDI, (mean ± S.D., n = 3)........ S2
Figure S2. The influence of the quantities of LAP on the DL, Ps, EE and PDI, (mean ± S.D., n = 3). S3
Figure S3. The influence of the quantities of TPGS on the DL, Ps, EE and PDI, (mean ± S.D., n = 3). S4
Figure S4. The effects of CUR, LAP, and TPGS on the DL in contour and surface plots. S5
Figure S5. The effects of CUR, LAP, and TPGS on the EE in contour and surface plots. S5
Figure S6. The effects of CUR, LAP, and TPGS on the Ps in contour and surface plots. S6
Figure S6. The effects of CUR, LAP, and TPGS on the PDI in contour and surface plots. S6

Experimental Section

1. The Single factor Experiments

In risk assessment, the level of risk may not always be documented in the existing literature. In such instances, it is crucial to conduct preliminary experiments to ascertain the risk more accurately. Single factor experiments provide a straightforward and convenient approach to evaluate the influence of various factors. Through these experiments, we can clearly establish the approximate range of factors that impact quality objectives. In this study, we employed single-factor experiments to identify the significant factors affecting the preparation process and prescription stability, thereby determining each factor and its corresponding level range for the optimization experiment. In the single-factor investigation of the process experiment, the primary focus is on the method of incorporating excipients, namely LAP and TPGS, alongside CUR. Several factors must be considered when forming LAP nanoparticles, including the ionic strength of the solution, the concentration of LAP, and the dissolution characteristics of the drug. During the process inspection, we examined various methods of adding these three materials, which established an initial basis for determining the optimal amounts of each component in the formulation. Following the determination of the process parameters, we conducted a separate investigation into the effects of varying quantities of LAP, TPGS, and CUR on the PS, PDI, DL, and EE of CUR-LAP-TPGS nanogel. Based on the results of this single-factor experiment, we subsequently optimized the formulation.

2. Effect of Control Factors on the Response

Contour and surface plots are utilized to analyze the effects of X on Y in CUR-TPGS-LAP nanogels. Here, X represents three influencing factors (independent variables): CUR, LAP, and TPGS, while Y corresponds to four response variables: DL, EE, Ps, and PDI. Contour and surface plots effectively illustrate the impact of two variables on a specified response variable while maintaining one variable constant. In this context, we fix the central point as a constant; for instance, when examining the effects of CUR and LAP on the response variables, TPGS is held constant at 30 mg. Conversely, when investigating the effects of TPGS and LAP on the response variable, CUR is fixed at 6 mg, and when studying the effects of TPGS and CUR on the response variable, LAP is fixed at 6.25 mg. Based on these preset values, a 5-level high line was selected for the output of the response variables, leading to the generation of contour and

surface plots (Figures S4, S5, S6, and S7).

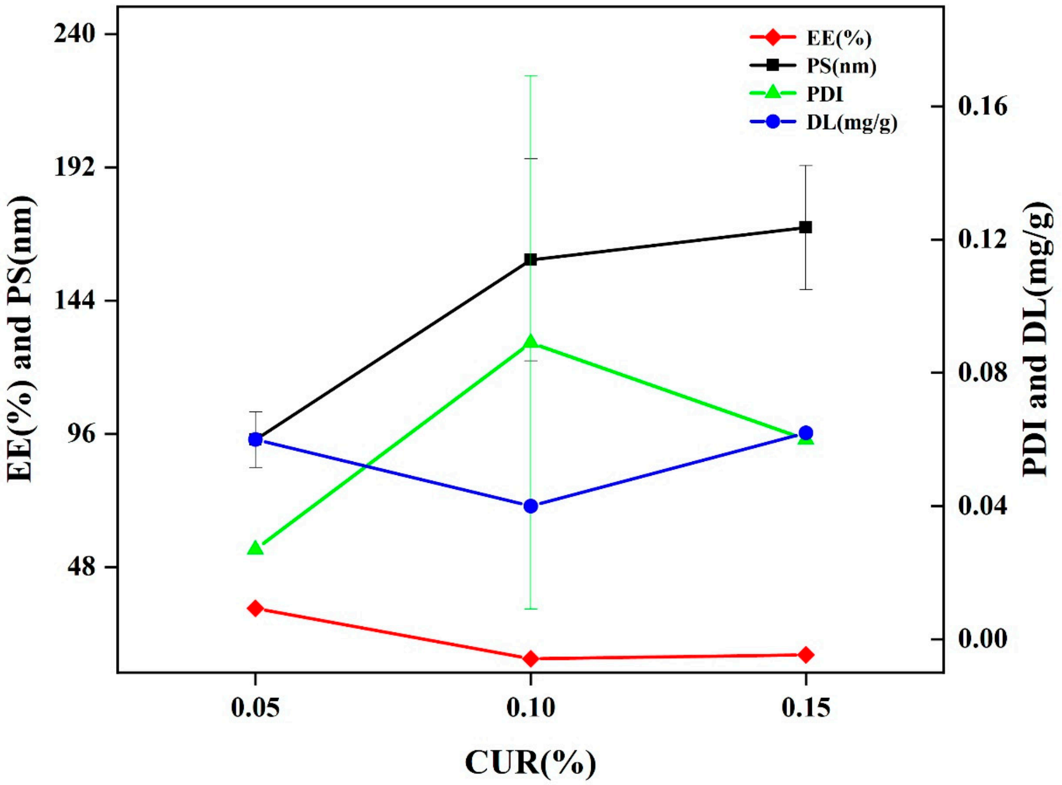

Figure S1. The influence of the quantities of CUR on the DL, Ps, EE and PDI, (mean ± S.D., n = 3).

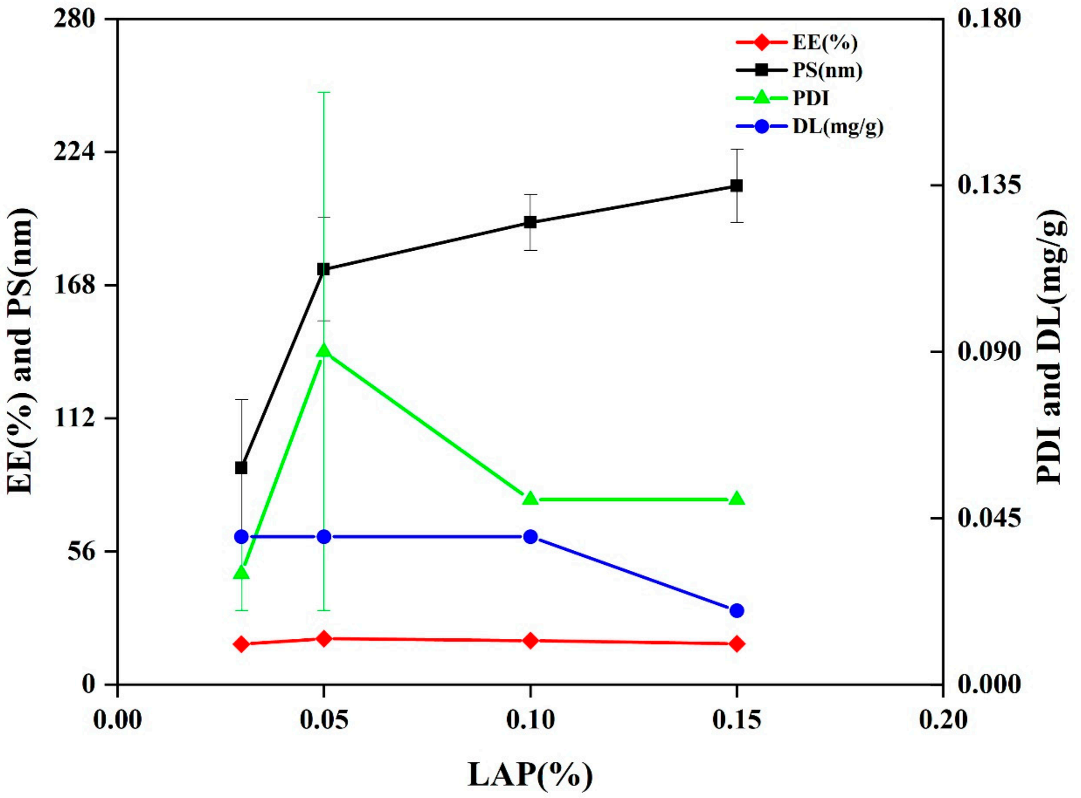

Figure S2. The influence of the quantities of LAP on the DL, Ps, EE and PDI, (mean ± S.D., n = 3).

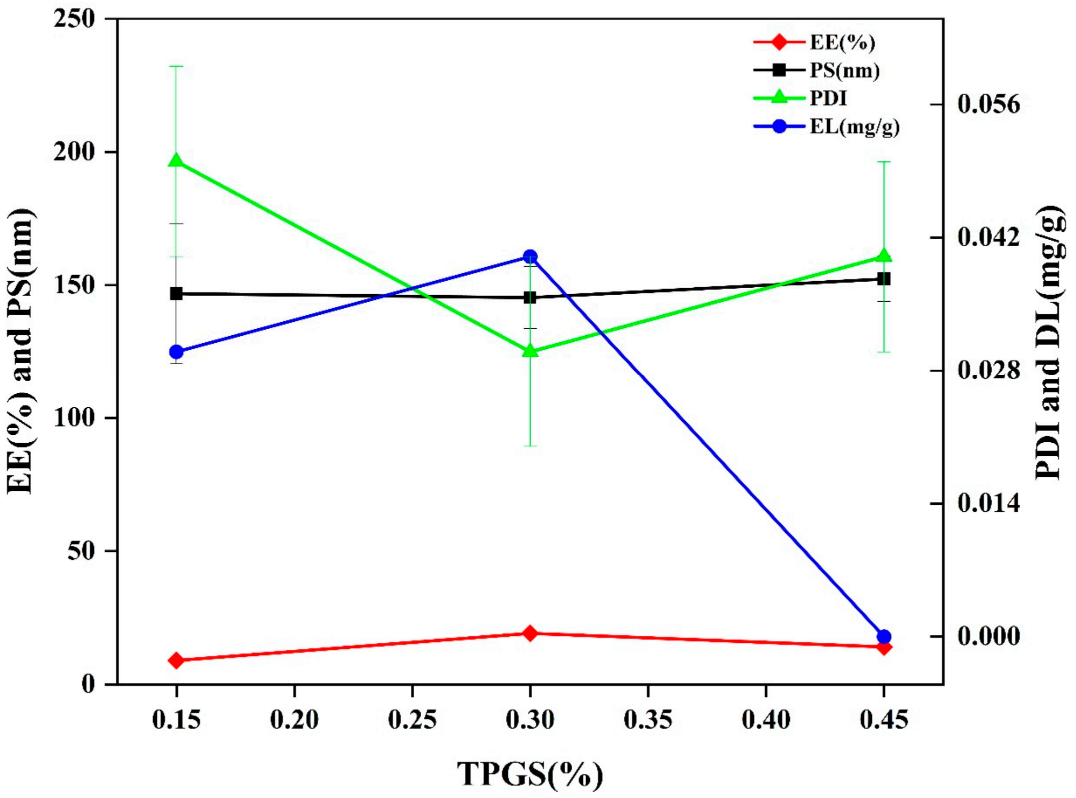

Figure S3. The influence of the quantities of TPGS on the DL, Ps, EE and PDI, (mean ± S.D., n = 3).

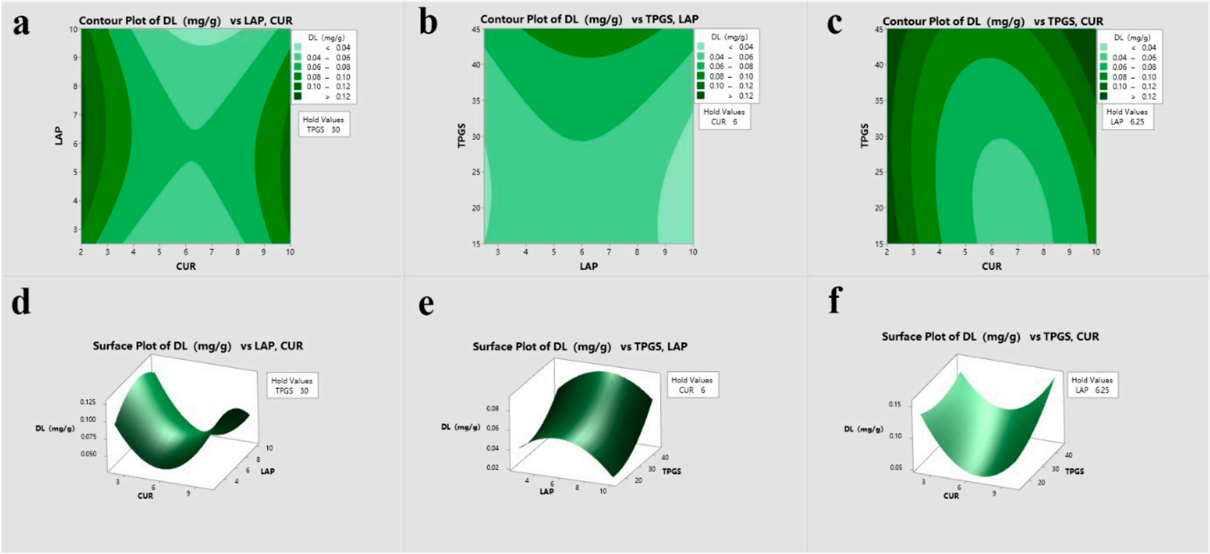

Figure S4. The effects of CUR, LAP, and TPGS on the DL in contour and surface plots.

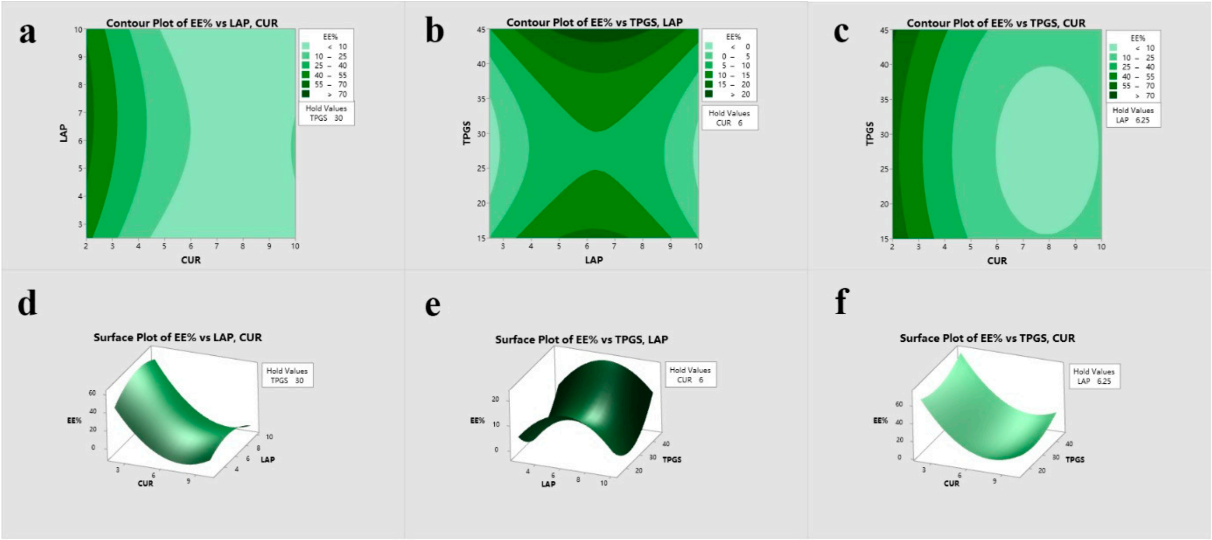

Figure S5. The effects of CUR, LAP, and TPGS on the EE in contour and surface plots.

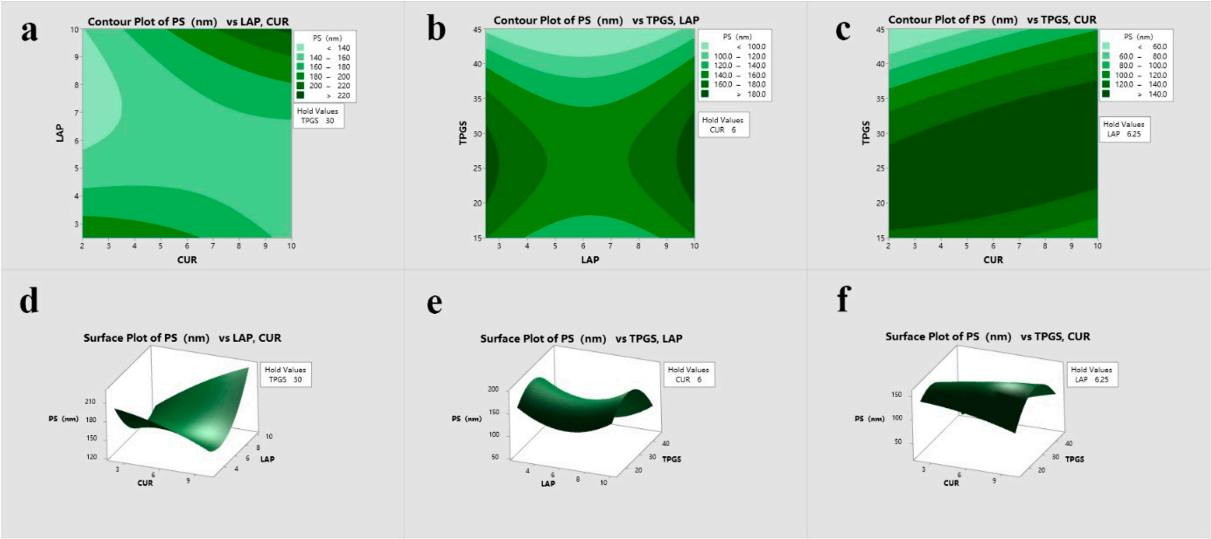

Figure S6. The effects of CUR, LAP, and TPGS on the Ps in contour and surface plots.

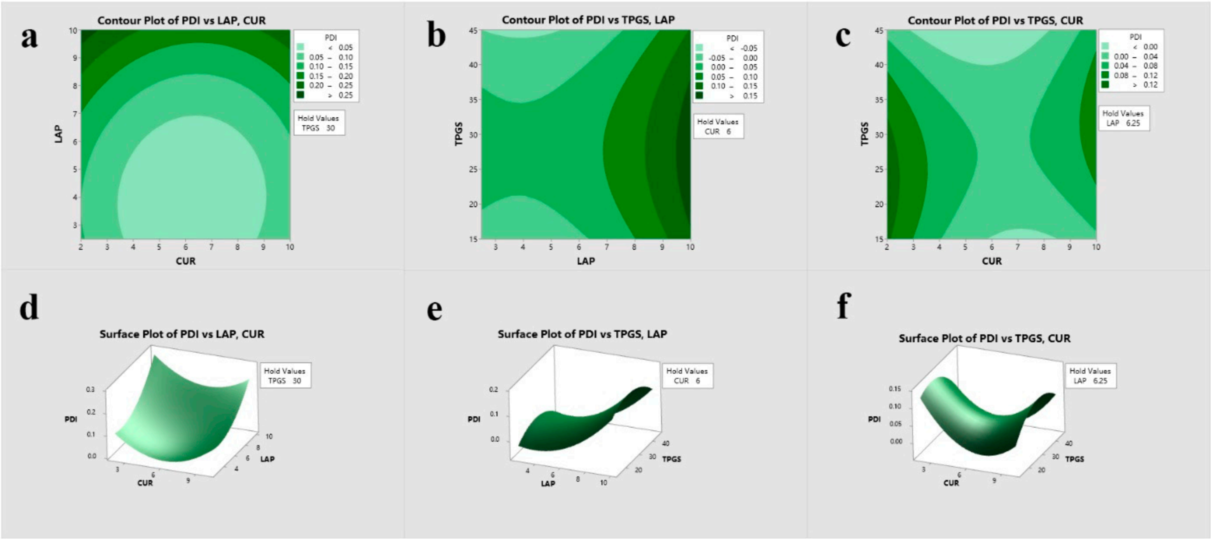

Figure S7. The effects of CUR, LAP, and TPGS on the PDI in contour and surface plots.
